# Periodontal management in patients with diabetes: the impact of periodontal treatment on cardiometabolic biomarkers

**DOI:** 10.3389/froh.2026.1922284

**Published:** 2026-08-27

**Authors:** Alicia Morales, Gustavo Sáenz-Ravello, Loreto Matamala, Mauricio Baeza, Patricia Hernández-Ríos

**Affiliations:** 1 Department of Conservative Dentistry, Faculty of Dentistry, Universidad de Chile, Santiago, Chile; 2 Centro de Epidemiologia y Vigilancia de las Enfermedades Orales (CEVEO), Faculty of Dentistry, Universidad de Chile, Santiago, Chile; 3 Interuniversity Center for Healthy Aging (CIES), Consortium of State Universities (CUECH), Santiago, Chile

**Keywords:** biomarkers, C-Reactive protein, diabetes mellitus, glycated hemoglobin, periodontal diseases, periodontitis, root planning

## Abstract

**Introduction:**

Type II Diabetes Mellitus (T2DM) and periodontitis present an independent bidirectional relationship. While T2DM is associated with a higher prevalence, incidence, and severity of periodontitis, periodontitis might worsen glycemic control and inflammatory biomarker levels in diabetics. Although there is no standardized therapy for the treatment of diabetic patients with periodontitis, different periodontal interventions report improvements in clinical and cardiometabolic parameters.

**Objective:**

To summarize the current evidence regarding the clinical management of patients with T2DM and periodontitis, with a secondary focus on the impact of periodontal interventions on cardiometabolic biomarkers.

**Materials and methods:**

A literature search was conducted between 2010 and 2026. Information about the clinical management of periodontitis in individuals with T2DM and its impact on cardiometabolic biomarkers was classified and summarized.

**Results:**

A comprehensive periodontal examination and diagnosis should be performed, with close collaboration with the patient's physician. Prior dental treatment, capillary blood glucose between 70 and 180 mg/dL, and up to 240 mg/dL in emergency procedures are suggested. The EFP's stepwise treatment approach is recommended, considering periodontal therapy in multiple sessions. Antibiotic therapy or prophylaxis are not recommended in all cases, whereas periodontal maintenance may be required every 3–6 months. Periodontal interventions in diabetic individuals have been shown to decrease glycated hemoglobin (HbA1c) and C-reactive protein (CRP), together with other inflammatory and cardiometabolic biomarkers.

**Conclusions:**

Patients with T2DM benefit from several periodontal treatment strategies, with a positive impact on inflammatory and cardiometabolic biomarkers associated with eventual reductions in diabetic control and systemic inflammation.

## 1 Introduction

Type 2 diabetes mellitus (T2DM) is a multisystemic noncommunicable disease (NCD) related to impaired insulin uptake or secretion, associated with chronic hyperglycemia that may lead to macrovascular, microvascular, and non-vascular complications such as neuropathy, glaucoma, and renal papillary necrosis, among others. T2DM is associated with high mortality and morbidity rates, has a negative impact on quality of life, and is included among the 2030 Global Targets established by the World Health Organization (WHO) (1, 2). Periodontitis, on the other hand, is a chronic, multifactorial inflammatory disease triggered by a dysbiotic biofilm and characterized by progressive destruction of the supporting tissues of the teeth (3). These two associated NCDs share common etiopathogenic pathways, social determinants, and risk factors, supporting the need to frame periodontal care not only as an isolated dental treatment, but also as part of broader NCD prevention and management (4). Both are prevalent public health problems worldwide, associated with other NCDs, such as cardiovascular diseases (CVDs), in observational and interventional studies (2, 5).

Substantial evidence suggests that the independent relationship between T2DM and periodontitis is bidirectional and causal (6, 7). While T2DM is associated with a higher incidence, prevalence, and severity of periodontitis (8, 9), severe periodontitis is linked to a worse diabetic status. Chronic hyperglycemia may impair host immune response, wound healing, and periodontal stability, while periodontal inflammation may contribute to an elevated inflammatory burden and poorer metabolic control (10). Uncontrolled diabetic patients show impaired circulating inflammatory and cardiometabolic biomarkers (11), higher levels of glycated hemoglobin (HbA1c), as well as greater T2DM complications (retinopathy, renal complications, neuropathic foot ulcers, cardiovascular mortality, coronary artery disease, cerebrovascular events, and subclinical coronary artery disease) (12–14).

Although there is no standardized therapy for the treatment of diabetic patients with periodontitis, different periodontal interventions report improvements in several clinical and cardiometabolic parameters.

This mini-review aimed to summarize the current evidence regarding the clinical management of patients with T2DM and periodontitis, with a secondary focus on the impact of periodontal interventions on cardiometabolic biomarkers.

## 2 Materials and methods

A search of the current scientific literature available in English and Spanish was conducted in PubMed, Cochrane Library, SciELO, and national clinical guidelines, covering the period from 2010 to 2026. Recommendations based on primary and secondary sources (observational and interventional studies, systematic reviews, consensus reports, and clinical guidelines), including adult patients with periodontitis and T2DM, were considered. Data on the clinical management of periodontitis (periodontal diagnosis, periodontal treatment modalities, use of antibiotics, adjuvant, and maintenance therapy) in individuals with T2DM, and its impact on cardiometabolic biomarkers were classified, summarized and described narratively.

## 3 Clinical management of periodontitis in patients with T2DM

### 3.1 Integrated medical care and oral health education

In patients with T2DM, periodontal care should be regarded as part of a broader general and cardiometabolic risk management strategy rather than as an isolated dental intervention (10). Multidisciplinary collaboration with the medical healthcare team is essential to improve metabolic and periodontal outcomes (15–19).

Glycemic targets, as established by the physician, may vary between patients according to diabetes duration, risk of hypoglycemia, comorbidities, polypharmacy, and individual cardiovascular risk (16, 20).

A comprehensive medical history and periodontal examination must be performed to define periodontitis stage (I–IV), extension, and grade (A, B, or C) (21). HbA1c testing might be associated with periodontitis diagnosis, management, and progression, so it is currently considered to classify patients with periodontitis as grade A (normoglycemic/no diagnosis of diabetes), grade B (HbA1c < 7.0% in diabetics), and grade C (HbA1c ≥ 7.0% in diabetics) (22, 23).

Oral health education should be provided to all patients with T2DM and periodontitis. Interventions focused on self-care (24, 25), particularly those that enhance self-efficacy in toothbrushing, are associated with significant reductions in HbA1c (26, 27), as well as improved clinical periodontal parameters (24, 26) and oral health- related quality of life (26).

### 3.2 Mechanical periodontal therapy

Before and during periodontal therapy, several general and dental care recommendations should be considered (18, 28)

- Dental appointments should be scheduled in the morning. Patients should be advised to take their usual medication and eat their normal meals before the dental visit (18, 28).
- Patient anxiety should be managed to avoid blood glucose level disturbances and prevent metabolic emergencies, especially hypoglycemia (18, 28). Metabolic emergencies, in turn, must be recognized and managed.
- Capillary blood glucose should be measured before periodontal treatment. Safe ranges are 70 mg/dL (pre-meal) to 180 mg/dL (post-meal) (18, 28), while emergency dental procedures might be performed with capillary blood glucose levels up to 240 mg/dL (18, 28).
- A 0.12% alcohol-free chlorhexidine gluconate solution rinsing before dental procedures is recommended to reduce bacterial load (15 mL for 60 s) (17)
- Relevant drug interactions must be considered (18, 28).

For individuals with periodontal health or gingivitis (29), primary prevention measures for periodontitis should be undertaken (30). Periodontitis (3, 22) should be treated according to the European Federation of Periodontology (EFP) treatment guidelines for periodontitis stage I–III (31) and IV (32), including the restoration of lost occlusal functional units (33).

Scaling and root planning, performed in multiple sessions (e.g., per quadrant) or within 24 h (full mouth scaling and root planning; FMSRP), leads to significant improvements in clinical parameters in smoker and non-smoker patients with T2DM (34–36). However, compared with FMSRP, quadrant-based scaling and root planning have shown greater reductions in HbA1c levels at 3 months after treatment (37, 38). Therefore, multiple sessions or the conventional quadrant-based treatment approach is considered the preferred option for patients with periodontitis and T2DM.

### 3.3 Adjunctive periodontal treatments

In patients with T2DM and periodontitis, antibiotic prophylaxis is not required in most cases (39–41). However, antibiotics may be prescribed for patients with poor diabetic control (e.g., fasting plasma glucose levels >200 mg/dL), bad oral health status, and/or the necessity of invasive dental procedures (28, 42).

On the other hand, the use of systemic antimicrobial therapy as an adjunct to non-surgical periodontal therapy (NSPT) results in statistically significant but clinically irrelevant improvements in clinical periodontal parameters, like probing depth (−0.15 mm, *p* < 0.05), without relevant benefits in clinical attachment level or bleeding on probing (43). Amoxicillin plus metronidazole (500 mg of amoxicillin and 200 mg of metronidazole, 3-times daily for 7 days), in conjunction with non-surgical periodontal treatment, results in slight improvements in the percentage of sites with probing depths >6 mm in patients with uncontrolled 2TDM (44), therefore showing a weak recommendation as an adjunct to conventional periodontal treatment in this type of patient (45, 46). In light of the above, the indication for antibiotic therapy in patients with T2DM should be guided primarily by the severity of periodontitis rather than by the diagnosis of diabetes *per se*, considering that the indiscriminate or improper use of antibiotics could contribute to the development of bacterial resistance and other side effects, as well as interact with specific hypoglycemic medications (47).

The adjunctive use of local antibiotic therapy (46, 48), antioxidants (49–51), and diode laser therapy (52), might have beneficial effects on periodontal tissues and glycemic control; however, they are not currently recommended because the evidence is weak, with high heterogeneity between studies.

### 3.4 Supportive periodontal therapy (SPT)

SPT may be scheduled at 3–6-month intervals, depending on glycemic control, periodontitis severity, and periodontal therapy response (53). In the absence of periodontitis at baseline, an annual periodontal assessment may be recommended (19). During SPT, strict metabolic control must be maintained to reduce the risk of periodontal disease progression and long-term tooth loss (54). The clinical procedures outlined in the EFP clinical practice guidelines for the treatment of periodontitis stages I–III (31) and IV (32) should be followed.

## 4 Periodontal therapy and cardiometabolic biomarkers in patients with type 2 diabetes mellitus

Emerging evidence indicates that T2DM, periodontitis, and CVDs constitute a mutually reinforcing triad (55), underpinned by systemic inflammation, oxidative stress, microbial dysbiosis, and shared genetic susceptibilities (56). Diabetes and periodontitis present a synergistic interaction, where chronic hyperglycemia and periodontal inflammation jointly amplify systemic cytokine release, endothelial dysfunction, and atherosclerotic progression (55). Observational studies have shown that periodontal disease may impair general inflammatory burden and consequently increase circulating metabolic and cardiovascular risk biomarkers (57, 58).

The following section synthesizes the evidence on how periodontal therapy might modify specific cardiometabolic biomarkers in patients with T2DM, and critically appraises the magnitude, duration, and clinical relevance of these changes. Table 1 summarizes the main reported effects of periodontal therapy on cardiometabolic biomarkers.

**Table 1:** Summary of the main reported effects of periodontal therapy on cardiometabolic biomarkers in patients with type 2 diabetes mellitus.

Biomarker category	Biomarker	Main effects of periodontal therapy
Glycaemic biomarkers	HbA1c	Consistent reduction (0.3%–0.6%) following non-surgical periodontal therapy.
	Fasting plasma glucose	Small reductions, although findings remain inconsistent across meta-analyses.
	HOMA-IR	Possible improvement in insulin sensitivity; the evidence is limited by the small number of available studies.
Inflammatory biomarkers	CRP/hs-CRP	Consistent reduction following periodontal therapy, reflecting decreased systemic inflammatory burden.
	TNF-α	Significant reduction after intensive non-surgical periodontal therapy.
	IL-6	Reduction reported after treatment, although effect sizes are heterogeneous.
	IL-1β	No reproducible treatment effect has been demonstrated.
Lipid biomarkers	Total cholesterol	Short-term reduction reported; effect disappears by six months.
	Triglycerides	Short-term reduction reported; the effect is not maintained over time.
	HDL-C	No clinically meaningful improvement.
	LDL-C	No consistent improvement following periodontal therapy.
Vascular and emerging biomarkers	Systolic blood pressure	Small reductions have been reported after non-surgical periodontal therapy.
	Diastolic blood pressure	Evidence remains inconsistent.
	Flow-mediated dilation (FMD)	Improvement in endothelial function following intensive periodontal therapy.
	Soluble E-selectin	Reduction suggesting improved endothelial activation.
	Salivary CRP/active MMP-8	Promising biomarkers for chairside oral-systemic risk assessment; clinical validation is still lacking.

### 4.1 Glycemic biomarkers

HbA1c remains the most extensively investigated systemic outcome of periodontal therapy in individuals with T2DM. The most recent Cochrane review, which doubled the number of included trials relative to its previous version, reported an absolute HbA1c reduction of 0.43% three to four months after subgingival instrumentation (moderate-certainty evidence), 0.30% at 6 months (95% CI: −0.52 to −0.08; 12 studies, 1,457 participants) and 0.50% at 12 months (95% CI: −0.55 to −0.45), the latter derived from a single trial (59). Comparable estimates have been reported elsewhere: −0.64% at three months and −0.33% at six months (60), and weighted mean differences of −0.514 (95% CI: −0.730 to −0.298) and −0.548 (95% CI: −0.859 to −0.238) at three and six months, respectively (61). The single largest 12-month randomized trial to date found that intensive periodontal treatment reduced HbA1c by 0.6% more than control periodontal care (*p* < 0.0001), alongside reductions in CRP and TNF-α (62).

Evidence for other glycemic indices is considerably weaker and less consistent. One meta-analysis reported a significant reduction in fasting plasma glucose of 8.95 mg/dL (95% CI: 4.30–13.61) following non-surgical therapy (63), whereas a Bayesian network meta-analysis found no advantage of scaling and root planning over no treatment for this outcome (MD: 4.91 mg/dL, 95% CI: −1.95 to 11.78) (64). Data on insulin resistance are scarcer still: a systematic review identified only seven eligible studies reporting HOMA-derived indices, of which four could be pooled, precluding firm conclusions (65). Fasting plasma glucose and HOMA-IR should therefore be regarded as exploratory rather than established outcomes of periodontal intervention.

### 4.2 Acute-phase and inflammatory biomarkers

Low-grade systemic inflammation (LSI) constitutes one of the most relevant links between periodontitis and other NCDs, like T2DM and CVDs (61, 62).

CRP is LSI most consistently responsive systemic marker (58, 66), and a validated cardiovascular risk marker, that stratifies cardiovascular risk into low, medium, and high (hsCRP: <1.0, 1.0–3.0, and >3.0 mg/L, respectively) (67). Pooled reductions of 0.52 mg/L at three months and 1.24 mg/L at six months after periodontal therapy have been reported (60), and a meta-analysis restricted to individuals with T2DM found a mean difference of −1.28 mg/L (95% CI: −2.07 to −0.48) favoring periodontal intervention (68) Barbirato et al. reported moderate-certainty evidence of a favorable effect on serum CRP/hs-CRP even in the absence of adjunctive antimicrobial therapy (69). The magnitude of these changes is clinically meaningful when interpreted against the cardiovascular risk strata described above (67).

TNF-α shows a comparable direction of effect, with pooled mean differences of −1.33 pg/mL (95% CI: −2.10 to −0.56) (68) and −1.90 pg/mL (95% CI: −3.05 to −0.74) at six months (70). A recent meta-analysis with spline-based meta-regression confirmed a significant post-treatment reduction in TNF-α (SMD: −0.59, 95% CI: −1.02 to −0.16) favoring intensive non-surgical therapy, and characterized the temporal kinetics of TNF-α, IL-1β, IL-6, and hs-CRP in the weeks following instrumentation (71). Reductions in IL-6 and systolic blood pressure have likewise been documented after non-surgical periodontal therapy in broader periodontitis populations (72, 73), whereas effects on IL-1β and diastolic blood pressure remain inconsistent.

### 4.3 Lipid profile

Evidence on lipid parameters is both more limited and more time-dependent. In a meta-analysis restricted to individuals with T2DM, periodontal therapy was associated with significant reductions in total cholesterol (MD: −0.47 mmol/L, 95% CI: −0.75 to −0.18) and triglycerides (MD: −0.20 mmol/L, 95% CI: −0.24 to −0.16) at three months, whereas HDL cholesterol changed marginally in favor of the control arm (MD: 0.06 mmol/L). Notably, no statistically significant difference was observed for any lipid fraction at six months (74). This attenuation contrasts with the trajectory described for CRP and suggests that lipid responses, being downstream of hepatic acute-phase signaling and strongly conditioned by concomitant lipid-lowering pharmacotherapy, are unlikely to represent a robust target of periodontal intervention.

### 4.4 Vascular function and emerging biomarkers

Beyond circulating mediators, periodontal therapy has been associated with improvements in endothelial function. Intensive treatment produced an absolute increase in flow-mediated dilation (FMD) of 2.0% at 6 months, together with reduced soluble E-selectin, despite a transient rise in CRP and IL-6 at 24 h; a separate trial reported improvement in FMD from 6.1% to 9.8% at three months alongside a fall in CRP from 1.1 to 0.8 mg/L. Additional candidate markers spanning the periodontal–cardiovascular axis include soluble ICAM-1 andVCAM-1, myeloperoxidase, lipoprotein-associated phospholipase A2, and fibrinogen, although none has been validated as a stand-alone prognostic indicator. Chairside detection of active MMP-8 and salivary CRP represents a plausible route to point-of-care oral–systemic risk screening, consistent with the findings on GCF and serum CRP described above (75, 76).

### 4.5 Magnitude, duration, and clinical relevance

Interpreting these estimates requires an explicit benchmark. Adding a second hypoglycemic agent typically achieves an absolute HbA1c reduction of 0.4%0.9%, a range proposed as a reference for interpreting trial results (59); the pooled effect of periodontal therapy therefore lies at the lower boundary of pharmacological second-line efficacy. At the population level, a 0.2% lower mean HbA1c has been associated with 10% lower mortality over two to five years, although such observational estimates are susceptible to confounding (59). Similarly, a reduction of approximately 1 mg/L in hs-CRP may shift patients across established cardiovascular risk categories (67). Economic evaluations reinforce this reading, indicating that periodontal treatment in patients with T2DM may be cost-effective under a range of assumptions (77–79).

## 5 Discussion

The management of patients with T2DM and periodontitis should be based on a comprehensive, multidisciplinary approach. To guarantee the safety and effectiveness of treatment, collaboration with the rest of the medical care team is essential. Oral health education (24, 26, 27), combined with multiple sessions of mechanical periodontal therapy (34, 35) and regular periodontal maintenance (54), may favor periodontitis stabilization, long-term tooth preservation, reduced systemic inflammation, and improved glycemic control (19, 80). Even in the absence of a gold standard protocol for periodontal care in patients with T2DM, diabetic patients benefit from different periodontal treatment strategies (35, 36, 53), with a reported improvement in oral clinical and cardiometabolic parameters (34).

However, these findings should be interpreted with caution, and future studies are necessary to determine the true effect of periodontal therapy on the progression and clinical outcomes of cardiovascular diseases (81). The magnitude of the effects of periodontal treatment on cardiometabolic biomarkers varies according to the follow-up period, biomarker type, study design, and study heterogeneity. For example, the beneficial effects of periodontal therapy on CRP levels are sustained or even increased after 6 months in some studies, whereas the effects of periodontal treatment on lipid profiles are no longer evident over the same period (59). Additionally, reductions in HbA1c following periodontal interventions decline before 6 months in some studies but are maintained at 12 months in others, with greater reductions observed in individuals with poorer baseline glycemic control (61). Findings regarding fasting plasma glucose, however, remain inconsistent (63).

Treatment protocols differ substantially between studies, and the NSPT modality selected may also have influenced the outcomes. FMSRP elicits a greater acute-phase response than quadrant-wise therapy. Individuals with a greater initial acute-phase response show smaller HbA1c reductions at three months (37, 38). Therefore, multiple-session quadrant-based therapy may be preferable in this population.

Finally, heterogeneous periodontitis case definitions, the absence of blinding, unreported units for pooled biomarker estimates, and residual confounding by smoking, obesity, and medication changes during follow-up limit comparability. Taken together, the evidence supports modest early improvements in surrogate biomarkers, with uncertain long-term durability and no demonstrated benefits on hard diabetic or cardiovascular outcomes to date. Long-term randomized trials using standardized periodontal protocols and harmonized biomarker measurements are still needed to determine whether improvements in surrogate markers translate into reductions in diabetic or cardiovascular complications (71–73).

On the other hand, a paradigm shift in the management of patients with periodontitis and T2DM should be revisited, from isolated procedure-driven care toward prevention-oriented, integrated, and value-based models. In this context, periodontal therapy should be incorporated into broader NCD strategies through risk-stratified care, supportive maintenance, medical–dental coordination, and prioritization of interventions with meaningful clinical and patient-centered benefits. Such an approach may improve oral and systemic outcomes while contributing to more equitable and sustainable healthcare delivery (77–79).
